# Health behaviour and its determinants in elderly patients with chronic diseases: evidence from Jiangsu Province, China

**DOI:** 10.1186/s12877-022-03010-w

**Published:** 2022-04-07

**Authors:** Li Chen, Yinghua Gong, Liang Yuan

**Affiliations:** 1 School of Economics, Changzhou University, Changzhou, 213000 People’s Republic of China; 2 Shanghai National Accounting Institute, Shanghai, 201799 People’s Republic of China; 3 College of Business, Shanghai University of Finance and Economics, Shanghai, 200433 People’s Republic of China

**Keywords:** Elderly patient, Chronic disease, Health behaviour, Determinants, Jiangsu Province

## Abstract

**Background:**

Chronic disease is a major cause of mortality among elderly individuals in China, and treatment is a substantial public health burden. However, behavioural interventions may be more important than mere clinical treatment of these chronic diseases.

**Objective:**

The paper aimed to assess the health behaviour of a sample of elderly individuals with chronic diseases in Jiangsu Province, China and to identify how demographic characteristics influence health behaviour. Furthermore, the group that would likely need the most health intervention was identified.

**Design:**

A version of the Health Promoting Lifestyle Profile II (HPLP-II) was adapted to Chinese to evaluate health behaviours in six dimensions: nutrition, tobacco and alcohol use, physical activity, stress management, health responsibility, and spiritual growth. Multistage random sampling was conducted from October 2020 to May 2021. Questionnaires incorporating the adapted HPLP-II were distributed to 900 elderly patients (i.e., aged 60 and above) with chronic diseases in the three sampled prefectures of Jiangsu Province; of these questionnaires, 791 were completed. Univariate t tests, principal component analysis, and multivariate linear regressions were employed in the analysis.

**Results:**

The average total score of respondents on health behaviour was 73.73. The dimensions (ordered from highest to lowest scores) are as follows: “nutrition”, “tobacco and alcohol use”, “health responsibility”, “spiritual growth”, “stress management”, and “physical activity”. The multivariate linear regression suggested that the determinants (*P* < 0.05) of health behaviour (total score) were income, sex, age, relationship status, residence, and education.

**Conclusions:**

Elderly patients with chronic diseases in Jiangsu Province generally behaved in a healthy manner. “Physical activity”, “stress management”, and “spiritual growth” were the dimensions that would most benefit from health intervention, while elderly single/divorced/widowed patients with lower income and less education should be the target group for health intervention.

## Introduction

Chronic noncommunicable diseases (NCDs), defined as diseases lasting one year or more with no evident communicable biological cause, include cardio-cerebrovascular disease (e.g., high blood pressure, stroke, coronary disease), cancer, chronic respiratory disease, and diabetes [4]. In 2016, NCDs led to 41 million deaths around the world, accounting for 71% of global mortality [37]. In China, NCD has become the main cause of death, especially among elderly residents [26].

As proposed in the literature, health behaviour, defined as a series of actions taken by an individual that affect health or mortality [28], is crucial to the treatment of NCD [2]. However, health behaviour in ageing individuals with NCDs has received less attention. Thus, this research intended to explore the health behaviour of elderly patients with NCDs.

Jiangsu Province, P. R. China was selected the sample region. This province contains an ageing population, with more than 18.50 million elderly residents (i.e., aged 60 and above), accounting for 21.84% of the whole population, and 2.8 million senior elderly residents (i.e., aged 80 and above), accounting for 15% of the elderly population. Notably, 77.4% of the elderly population in Jiangsu Province suffers from NCDs [16].

According to the Health Promotion Model [27], an individual regulates her behaviour based on her characteristics, experience, and knowledge. This research aimed to identify the factors that influence the health behaviour of elderly patients with NCDs. In addition, this paper aimed to identify typical health behaviour so that the target group for health intervention could be determined. Furthermore, we categorized health behaviour into six dimensions and identified the determinants of health behaviour at a dimensional level. Therefore, we identified the dimensions in need of health intervention.

## Method

### Sample

The survey was conducted in Jiangsu Province from October 2020 to May 2021. The “ageing ratio” (i.e., ratio of the population aged 60 and above compared to the whole population) in this province ranks sixth among the 32 province-level regions in mainland China and is 3.14% higher than the average ageing ratio across the country. Compared with other provinces in China, the ageing population in Jiangsu Province is not only more striking but also more complex. The 13 prefectures in this province are traditionally divided into three regions in terms of geography and economy: the northern (5 prefectures, with a CDP per capita of 79,568 CNY in 2020), central (3 prefectures, with a CDP per capita of 127,357 CNY in 2020), and southern (5 prefectures, with a CDP per capita of 156,393 CNY in 2020) regions. In the latter two regions, residents aged over 60 years accounted for more than a quarter of the population in 2020, while the ageing ratio of the entire province was 21.84% [16]. In terms of the ageing ratio in each prefecture, Nantong (a central prefecture) ranks highest (30%), followed by Zhenjiang (a southern prefecture), Taizhou (a central prefecture), Wuxi (a southern prefecture), and Yangzhou (a central prefecture). One prefecture was selected from each region using simple random sampling; thus, the sampled prefectures of this survey are Wuxi (southern), Nantong (central), and Xuzhou (northern).

**Fig. 1:**
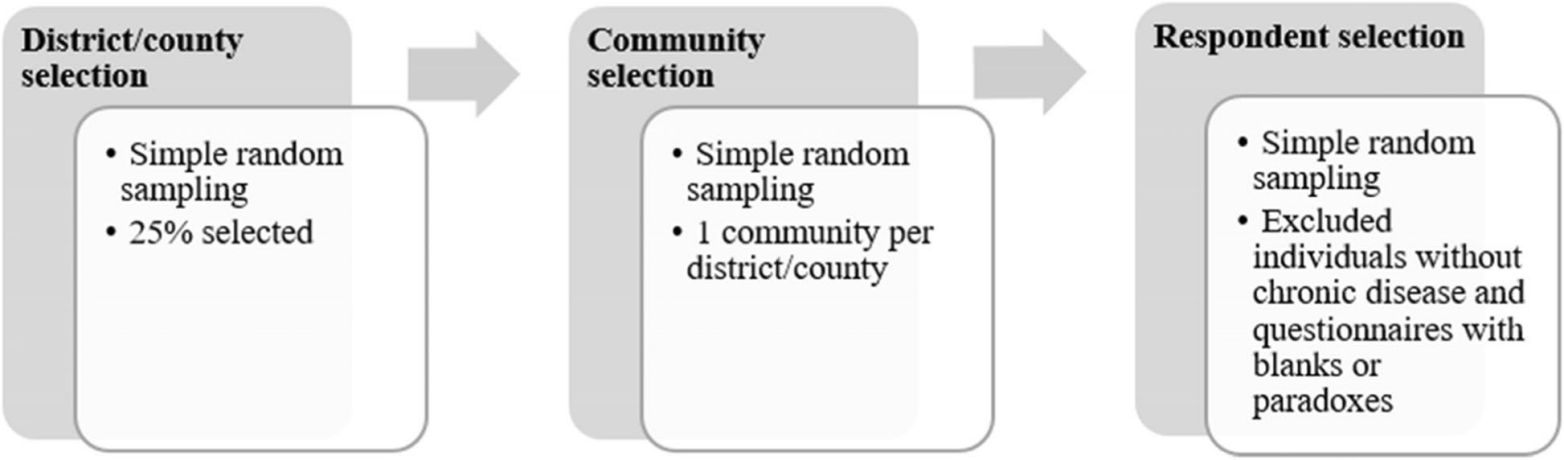
Sampling process

We employed multistage random sampling. In Stage 1, districts/counties^1^ were selected using simple random sampling, for a total of 25% of districts and 25% of counties. In Stage 2, communities were selected using simple random sampling, with one community selected for each district/county in the sample. In Stage 3, respondents were selected using simple random sampling. The number of respondents in each sampled district/county was proportionate to the population of the district/county. Residents aged over 60 that provided consent in the sample prefectures were potential. Residents who met these criteria but were unable to communicate because of muteness or other diseases were excluded from this survey.^2^ Thus, three hundred and ten questionnaires were distributed in each sample prefecture for a total of 930 distributed questionnaires. Questionnaires returned with blanks^3^ or logical paradoxes were excluded, resulting in 791 valid questionnaires (an effective recovery rate of 87.89%). Figure 1 shows how the 791 valid questionnaires were selected.

### Questionnaire

The questionnaire consisted of two parts: A) the sociodemographic profile and B) the health behaviour of elderly patients with NCDs. According to the literature, the variables in Part A that were directly or indirectly related to individual health behaviour included sex, age, residence, relationship status, location, education, income, and NCD category (i.e., cardio-cerebrovascular disease such as high blood pressure, stroke or coronary disease; cancer; chronic respiratory disease; or diabetes). Part B was a series of scales based on the Health Promoting Lifestyle Profile II (HPLP-II), with the 52 items simplified into 25 items for this survey (see Sect. 2.3 for more details).

### Instrument

#### Adapted version of HPLP-II

The HPLP-II [33], a revised version of the HPLP [34], is widely used to measure health-promoting lifestyles through a set of 52 items. As the target respondents were elderly residents in mainland China, the HPLP-II was adapted for this location. For example, substance abuse (i.e., smoking and excessive alcohol consumption) was introduced. Smoking and drinking are prevalent among elderly residents in China [20][36] and are associated with socioeconomic status [36], health literacy [24], psychological pressure [23], and activities of daily living (ADLs) [10]. In addition, evidence shows that a NCD diagnosis mitigates behaviours such as smoking and drinking [38]. Specifically, the adapted version of the HPLP-II employed in Part B of the questionnaire included 6 dimensions of health behaviours: nutrition (i.e., consuming grains, fresh fruit and vegetables, and protein as well as eating breakfast and maintaining sanitary food preparation), tobacco and alcohol use (i.e., smoking and excessive alcohol consumption); physical activity (i.e., daily exercise such as going for walks, entertaining exercise such as group dancing, and outdoor activity such as hiking); stress management (i.e., communication with spouse, communication with children, social activities, adequate sleep, and interpersonal relationship); health responsibility (i.e., daily tooth brushing, regular physical examinations, health knowledge, obtaining timely medical treatment, and following doctors’ advice); and spiritual growth (i.e., optimism, clear goals, confidence in the future, and happiness in daily life).

#### Scale

In Part B of the questionnaire, a 4-point Likert scale was employed, where 1 denoted “never” (i.e., the respondent never displays such behaviour), 2 denoted “sometimes” (i.e., the respondent has displayed such behaviour several times), 3 denoted “often” (i.e., the respondent displays such behaviour in most cases), and 4 denoted “routinely” (i.e., the respondent displays such behaviour almost all the time). Notably, items in the dimension of “tobacco and alcohol use” were reverse-scored, indicating unhealthy behaviours. Therefore, the score in this dimension was subtracted from 5, and the remainder was used as the final score. Scores on each dimension were a subtotal of the corresponding items, and the total score on the questionnaire was the total of all dimensions, with possible scores ranging from 25 to 100. Higher scores indicate healthier behaviour. Similar to the HPLP-II, this survey divided respondents into four levels of health promotion: bad (scoring 1–24), moderate (scoring 43–62), good (scoring 63–80), and excellent (scoring 81–100).

### Data management & analysis

#### Validity

The questionnaire was examined by five experts to determine its content validity index (CVI) according to three criteria (i.e., simplicity, relevance, and clarity). A four-point Likert scale was used to evaluate these three criteria, and items with a score below 0.75 were removed. Furthermore, the adapted version was pilot tested with a convenience sample of 30 respondents to assess the face validity. Final revisions were made accordingly. The Chinese version of the 36-item Short Form Health Survey (SF-36) developed by L. Li, Wang, and Shen [22] was used to check the criterion validity. The Chinese version of the SF-36 is used to assess health-related quality of life, and its reliability and validity have been tested with the general population in mainland China. The total scores of these two questionnaires were significantly positively related, with *r* = 0.32 and *P* < 0.001.

#### Quality control

A major concern in self-reported data is impression management bias [7]. For example, people under-report drinking frequency in English-speaking countries [29]. The reliability and validity of the self-report method are highly dependent on the characteristics of respondents as well as the cross-check approach [9] because alcohol consumption is related to culture and lifestyle [21]. To address this concern, we required a caregiver to be present as the survey was conducted, to justify collecting self-report data. Furthermore, ad hoc training was conducted to ensure that all investigators were qualified to explain the questionnaire in plain words to the respondents. As the survey was carried out, a random 10% of daily records were checked to detect and correct any omissions or typos in a timely manner. Data input was double-checked and logically tested. Questionnaires without key variables (e.g., sex, age) were excluded.

#### Software & statistics

The statistical analysis followed the five steps explained below, employing SPSS 17.0 software.

First, descriptive statistics were used to analyse the demographic characteristics of respondents.

Second, dimensional scores were standardised using Eq. (1).

1 $${Score}_{i}^{*}=\frac{{Score}_{i}}{{Full}_{i}}\times 100$$

where ${Score}_{i}^{*}$ denotes the standardised score on dimension $i$; ${Score}_{i}$ denotes the observed score on dimension $i$; and ${Full}_{i}$ denotes the highest possible score on dimension $i$.

Third, two sets of t tests were employed to determine the demographic characteristics that significantly impacted health behaviour. The first set of t tests captured the impact of demographic characteristics on health behaviour as a whole. Thus, the dependent variable was the total health behaviour score, and the independent variables were the demographic characteristics (i.e., variables collected in Part A of the questionnaire). The second set of t tests captured the impact of demographic characteristics on health behaviours in each dimension. Thus, the dependent variables were the dimensional scores on health behaviour, and the independent variables were the same as those in the first set of t tests. Demographic variables that were significant (*P* value < 0.05) in terms of mean value were considered factors that exerted a significant influence on health behaviour.

Fourth, principal component analysis (PCA) was conducted to extract the principal components of the demographic variables in each health-behaviour dimension. Demographic variables with eigenvalues greater than 1 were considered principal components. Furthermore, we conducted a robustness test for the results of the univariate t test by comparing the principal components and the factors identified by the univariate t test. Thus, variables that showed nonsignificant differences on the univariate t test were excluded from the multivariate regression.

Fifth, multivariate regression was used to capture the final determinants of health behaviour for the target group, where the total and dimensional health-behaviour scores were the dependent variables and demographic characteristics that were significant according to the univariate t test were the independent variables.

## Results

### Demographic characteristics of respondents

Table 1 shows the demographic characteristics of the 791 respondents. There were slightly more urban residents (58%) than rural residents (42%). The respondents were equally distributed in the southern (39%), central (30%), and northern (31%) regions. Most respondents (93%) had an upper-middle or above income (i.e., the average individual annual income of the household was more than 10,000 CNY), while only 7% of respondents had a low-level income (i.e., the average individual annual income of the household was less than 10,000 CNY). There were slightly more female respondents (55%) than male respondents (45%). Senior elderly respondents (i.e., aged over 80) accounted for 15% of respondents above 60 years old. Of the respondents, 64% were married, and 36% were single, divorced, or widow(er)s. Only 29% of respondents lived with their children; the other 71% lived with their spouse or on their own. In terms of education, most respondents (89%) did not have a bachelor’s degree or above, which is consistent with the relatively low proportion (19.96%) of individuals with higher education in the population of the entire province. In terms of disease category, respondents predominantly (73%) had cardio-cerebrovascular diseases (e.g., high blood pressure, stroke, or coronary disease).

**Table 1:** Demographic characteristics of respondents

Variable	Response	Frequency	%
Area	Urban	441	58
	Rural	350	42
Region	Southern	308	39
	Central	237	30
	Northern	246	31
Average individual annual income of household	Lower-middle (10,000 CNY and below)	57	7
	Middle (10,000–30,000 CNY)	421	53
	Upper-middle (30,000 CNY and above)	313	40
Sex	Male	359	45
	Female	432	55
Age	60–80 years old	669	85
	> 80 years old	122	15
Marriage	Married	504	64
	Single/divorce/widowed	287	36
Residence	Lives with other(s)	560	71
	Lives alone	231	29
Education	Junior secondary school and below	326	41
	High school	376	48
	Associate bachelor’s degree and above	89	11
NCD category	Cardio-cerebrovascular disease (e.g., high blood pressure, stroke, or coronary disease)	576	73
	Metabolic disorder (e.g., diabetes, gout, or osteoporosis)	143	18
	Respiratory disease (e.g., chronic bronchitis or emphysema)	63	8
	Cancer	9	1

### Health-behaviour scores

Table 2 presents the health-behaviour scores of the 791 respondents. Respondents could score up to a total of 100 points; the average total score on the six dimensions was 73.73 ± 8.71. In terms of each dimension, respondents scored the highest on “nutrition” (82.6 ± 8.92, standardised), followed by “tobacco and alcohol use” (77.13 ± 12.63, standardised) and “health responsibility” (76.15 $\pm$ 12.85, standardised), while respondents scored the lowest on “physical activity” (70.17 ± 10.25, standardised).

**Table 2:** Health behaviour scores across the dimensions

Dimension	Possible score	Score range Mean $\pm$ S.D	Standardised score range Mean/Possible score	Rank
Nutrition	24	19.83 $\pm$ 2.14	82.63 $\pm$ 8.92	1
Tobacco and alcohol use	8	6.17 $\pm$ 1.01	77.13 $\pm$ 12.63	2
Physical activity	12	8.42 $\pm$ 1.23	70.17 $\pm$ 10.25	6
Stress management	20	15.04 $\pm$ 2.03	75.20 $\pm$ 10.15	5
Health responsibility	20	15.23 $\pm$ 2.57	76.15 $\pm$ 12.85	3
Spiritual growth	16	12.04 $\pm$ 2.12	75.25 $\pm$ 13.25	4
Total	100	76.73 $\pm$ 8.71	76.73 $\pm$ 8.71	N/A

Table 3 presents respondents’ scores for each item. Specifically, in the dimension of “nutrition”, respondents scored the highest on the item “grains” (96.75 ± 0.06, standardised), while they scored the lowest on “fresh fruit” (74.00 ± 0.09, standardised). In the “tobacco and alcohol use” dimension, respondents scored higher on the item “excessive alcohol consumption” than on “smoking”. In the dimension of “physical activity”, respondents scored the highest on the item “daily exercise” (78.25 ± 0.06, standardised) and the lowest on “outdoor activity” (54.50 ± 0.06, standardised). In the dimension of “stress management”, respondents scored the highest on the item “communication with spouse” (87.75 ± 0.07, standardised) and the lowest on “adequate sleep” (48.25 ± 0.08, standardised). In the dimension of “health responsibility”, respondents scored the highest on the item “daily tooth brushing” (92.00 ± 0.11, standardised) and the lowest on “obtaining timely medical treatment” (55.25 ± 0.27, standardised). Finally, in the dimension of “spiritual growth”, participants scored the highest on the item “optimism” (79.75 ± 0.05, standardised) and the lowest on having “clear goals” (67.75 ± 0.12, standardised).

**Table 3:** Health behaviour scores of respondents within dimensions

Dimension	Health behaviour	Possible score	Score range Mean $\pm$ S.D	Standardised score range	Within-dimension rank
**Panel A**					
Nutrition	Grains	4	3.87 $\pm$ 0.25	96.75 ± 0.06	1
	Fresh vegetables	4	3.52 $\pm$ 0.24	88.00 ± 0.06	2
	Fresh fruit	4	2.96 $\pm$ 0.37	74.00 ± 0.09	6
	Protein	4	3.01 $\pm$ 0.19	75.25 ± 0.05	5
	Eats breakfast	4	3.41 $\pm$ 0.26	85.25 ± 0.07	3
	Sanitary food practices	4	3.06 $\pm$ 0.31	76.50 ± 0.08	4
Tobacco and alcohol use	Smoking	4	2.85 $\pm$ 0.52	71.25 ± 0.13	2
	Excessive alcohol consumption	4	3.32 $\pm$ 0.39	83.00 ± 0.10	1
Physical activity	Daily exercise (e.g., going for a walk)	4	3.13 $\pm$ 0.25	78.25 ± 0.06	1
	Entertaining exercise (e.g., group dancing)	4	3.11 $\pm$ 0.24	77.75 ± 0.06	2
	Outdoor activity (e.g., hiking)	4	2.18 $\pm$ 0.24	54.50 ± 0.06	3
**Panel B**					
Stress management	Communication with spouse	4	3.51 $\pm$ 0.27	87.75 ± 0.07	1
	Communication with children	4	3.29 $\pm$ 0.32	82.25 ± 0.08	3
	Social activity	4	3.43 $\pm$ 0.24	85.75 ± 0.06	2
	Adequate sleep	4	1.93 $\pm$ 0.32	48.25 ± 0.08	5
	Interpersonal relationships	4	2.88 $\pm$ 0.19	72.00 ± 0.05	4
Health responsibility	Regular physical examinations	4	3.31 $\pm$ 0.31	82.75 ± 0.08	2
	Daily tooth brushing	4	3.68 $\pm$ 0.42	92.00 ± 0.11	1
	Health knowledge	4	3.05 $\pm$ 0.22	76.25 ± 0.06	3
	Obtaining timely medical treatment	4	2.21 $\pm$ 1.08	55.25 ± 0.27	5
	Following doctor’s advice	4	2.98 $\pm$ 0.37	74.50 ± 0.09	4
Spiritual growth	Optimism	4	3.19 $\pm$ 0.21	79.75 ± 0.05	1
	Clear goals	4	2.71 $\pm$ 0.46	67.75 ± 0.12	4
	Confidence in future	4	3.02 $\pm$ 0.53	75.50 ± 0.13	3
	Happiness in daily life	4	3.12 $\pm$ 0.29	78.00 ± 0.07	2

### Univariate analysis of health behaviour

Table 4 presents the results of the univariate t test. In this table, Column (1) displays the results of the first set of t tests, where the dependent variable was the total health-behaviour score and the demographic characteristics were the independent variables. As shown in Column (1), income, sex, age, relationship status, residence, and education had a significant influence on health behaviour (i.e., *P* value < 0.05). Columns (2) – (7) display the results of the second set of t tests, where we changed the dependent variable of each column to the dimensional health-behaviour score and retained the same independent variables as in Column (1). Specifically, the results in Column (2) indicate that “nutrition” is significantly (i.e., *P* value < 0.05) affected by sex, age, relationship status, and education; the results in Column (3) indicate that “tobacco and alcohol use” is significantly (i.e., *P* value < 0.05) affected by sex and NCD category; the results in Column (4) indicate that “physical activity” is significantly (i.e., *P* value < 0.05) affected by income, age, residence, and education; the results in Column (5) indicate that “stress management” is significantly (i.e., *P* value < 0.05) affected by income, age, relationship status, and residence; and the results in Columns (6) and (7) indicate that “health responsibility” and “spiritual growth” are significantly (i.e., *P* values < 0.05) affected by income, age, relationship status, residence, and education.

**Table 4:** Univariate analysis of health behaviour scores

Variable	N	Score	Nutrition	Tobacco and alcohol use	Physical activity	Stress management	Health responsibility	Spiritual growth
**Panel A**								
Urban	441	77.12$\pm$9.15	19.73$\pm$2.05	6.18$\pm$1.16	8.85$\pm$2.13	15.38$\pm$2.07	14.90$\pm$2.74	12.08$\pm$2.33
Rural	350	76.23$\pm$6.43	19.95$\pm$2.15	6.16$\pm$0.91	7.88$\pm$1.02	14.61$\pm$1.53	15.64$\pm$2.02	11.99$\pm$1.83
* t* value		0.41	1.43	-0.29	0.71	1.73	1.80	0.94
* P* value		0.586	0.197	0.641	0.484	0.069	0.071	0.349
Southern	308	77.18$\pm$8.74	19.84$\pm$1.62	6.18$\pm$0.94	8.61$\pm$1.15	15.17$\pm$1.52	15.29$\pm$1.30	12.09$\pm$2.71
Central	237	76.01$\pm$6.14	19.79$\pm$1.58	6.19$\pm$0.97	8.42$\pm$0.93	14.90$\pm$2.32	14.75$\pm$2.52	11.96$\pm$1.44
Northern	246	76.86$\pm$6.58	19.85$\pm$2.31	6.14$\pm$1.06	8.18$\pm$2.03	15.01$\pm$2.06	15.61$\pm$2.92	12.06$\pm$2.01
* F* value		2.28	1.74	2.31	2.15	2.23	1.31	7.62
* P* value		0.080	0.126	0.074	0.090	0.083	0.269	< 0.001
**Panel B**								
Lower-middle income level	57	71.73$\pm$10.43	19.79$\pm$2.16	7.66$\pm$1.91	7.12$\pm$0.93	12.60$\pm$2.44	14.76$\pm$2.13	9.79$\pm$1.82
Middle income level	421	76.08$\pm$8.15	19.81$\pm$2.01	7.16$\pm$0.92	7.92$\pm$1.02	14.80$\pm$1.69	14.93$\pm$1.48	11.46$\pm$2.60
Upper-middle income level	313	78.51$\pm$6.97	19.86$\pm$1.95	4.57$\pm$0.86	8.82$\pm$1.13	15.81$\pm$2.01	16.43$\pm$0.92	13.01$\pm$2.11
* F* value		7.68	2.75	1.65	6.13	5.80	5.47	11.84
* P* value		< 0.001	0.061	0.159	< 0.001	0.001	0.004	< 0.001
Male	359	76.47$\pm$10.12	19.66$\pm$2.85	5.99$\pm$1.02	8.51$\pm$0.93	15.25$\pm$2.13	15.12$\pm$2.70	11.94
Female	432	76.95$\pm$6.03	19.97$\pm$1.59	6.32$\pm$0.96	8.35$\pm$1.43	14.87$\pm$2.01	15.32$\pm$1.63	12.12
* t* value		-2.17	-2.09	3.91	0.40	4.18	1.90	-0.11
* P *value		0.031	0.042	< 0.001	0.572	< 0.001	0.064	0.902
Aged 60–80	669	78.43$\pm$6.18	19.99$\pm$1.69	6.17$\pm$0.93	8.62$\pm$1.13	15.08$\pm$2.32	16.51	12.06$\pm$2.15
Aged 80 and above	122	67.41$\pm$11.03	18.95$\pm$2.15	6.18$\pm$1.03	7.32$\pm$0.97	14.82$\pm$1.97	8.21	11.93$\pm$1.61
* t* value		3.87	3.51	0.81	2.97	4.21	7.38	4.45
* P* value		< 0.001	< 0.001	0.413	0.002	< 0.001	< 0.001	< 0.001
**Panel C**								
Married	504	79.87$\pm$9.05	19.95$\pm$1.89	6.19$\pm$0.87	8.49$\pm$1.01	15.45$\pm$1.31	15.71$\pm$2.78	14.07$\pm$2.29
Single/divorced/widowed	287	71.21$\pm$5.43	19.62$\pm$2.39	6.13$\pm$1.03	8.30$\pm$1.13	14.32$\pm$2.45	14.39$\pm$1.92	8.45$\pm$1.83
* t* value		4.37	7.72	1.51	1.44	5.13	6.02	7.08
* P* value		< 0.001	< 0.001	0.101	0.120	< 0.001	< 0.001	< 0.001
Lives with other(s)	560	75.91$\pm$8.12	19.81$\pm$1.69	6.18$\pm$0.92	8.22$\pm$1.24	14.87	15.18	11.65
Lives alone	231	78.73$\pm$8.73	19.89$\pm$2.09	6.15$\pm$1.31	8.91$\pm$0.93	15.45	15.35	12.98
* t *value		4.06	-1.94	0.89	3.85	4.81	3.95	5.74
* P* value		< 0.001	0.051	0.340	0.003	< 0.001	< 0.001	< 0.001
**Panel D**								
Junior secondary school and below	326	74.23$\pm$8.06	19.76$\pm$2.19	6.16$\pm$1.23	8.30$\pm$1.93	15.01$\pm$3.51	14.44$\pm$2.83	10.55$\pm$1.93
High school	376	77.83$\pm$5.13	19.86$\pm$1.99	6.17$\pm$0.81	8.47$\pm$0.98	14.93$\pm$1.23	15.54$\pm$2.47	12.86$\pm$2.03
Bachelor’s degree and above	89	81.24$\pm$11.24	19.96$\pm$1.71	6.19$\pm$1.02	8.65$\pm$1.01	15.61$\pm$1.94	16.81$\pm$1.63	14.02$\pm$0.63
* F* value		7.85	8.17	2.81	4.20	2.41	9.91	19.34
* P* value		< 0.001	< 0.001	0.060	0.006	0.081	< 0.001	< 0.001
**Panel E**								
Cardio-cerebrovascular disease	576	76.81$\pm$9.87	19.84$\pm$2.09	6.19$\pm$1.14	8.48$\pm$1.01	14.75$\pm$1.43	16.23$\pm$2.93	11.32$\pm$1.93
Metabolic disorder	183	77.19$\pm$5.76	19.81$\pm$1.84	6.11$\pm$1.01	8.60$\pm$0.73	16.48$\pm$2.43	11.30$\pm$1.81	14.89$\pm$2.03
Respiratory disease	63	75.06$\pm$5.22	19.79$\pm$2.79	6.11$\pm$1.01	7.48$\pm$0.93	14.20$\pm$1.88	15.01$\pm$2.01	12.47$\pm$2.28
Cancer	9	75.92$\pm$6.13	19.82$\pm$1.98	6.29$\pm$0.84	8.29$\pm$0.94	16.45$\pm$2.71	15.21$\pm$1.93	9.85$\pm$2.13
* F* value		1.84	1.91	3.14	0.87	1.24	2.25	2.91
* P* value		0.137	0.164	0.007	0.546	0.301	0.081	0.057

Notably, the demographic variables included in the principal components of the PCA were those that showed significant differences in the univariate t test in Table 4. Thus, we tested the robustness of the results of the univariate t test.

### Multivariate analysis of health behaviour

Table 5 presents the results of multivariate regression, wherein the total and dimensional health-behaviour scores were the dependent variables and the demographic characteristics that showed significant differences in the univariate t test were the independent variables. As shown in Table 5, the total health-behaviour score was significantly (i.e., *P* value < 0.05) affected by income, sex, age, relationship status, and education. The dominant determinants of “nutrition” were age and education; the dominant determinants of “tobacco and alcohol use” were sex and education; the dominant determinants of “physical activity” were age and residence; the dominant determinants of “stress management” were age and income; the dominant determinants of “health responsibility” were income and education; and the dominant determinants of “spiritual growth” were income, age, and education.

**Table 5:** Multivariate linear regression for health behaviour

Dependent variable (score)	Independent variable (score)	Group	Reference group	b (95% CI)	S.E	*t* value	*P*value
**Panel A**							
Health behaviour	Income	Middle	Lower-middle	3.83 (1.39, 7.21)	1.72	2.21	0.028
		Upper-middle		4.43 (1.42, 8.64)	2.12	2.18	0.031
	Age	80 and above	60–80	-3.57 (-5.48, -1.65)	0.96	-3.69	< 0.001
	Marriage	Single/divorced/widowed	Married	-4.41 (-6.44, -2.73)	0.94	-4.76	< 0.001
	Education	High school	Junior secondary school and below	4.12 (1.73, 6.53)	1.26	3.35	< 0.001
		Bachelor’s degree and above		3.37 (1.51, 6.47)	1.31	2.71	0.009
Nutrition	Age	80 and above	60–80	-0.51 (-0.82, -0.13)	0.17	-2.57	0.013
	Education	High school	Junior secondary school and below	0.64 (0.19, 1.13)	0.23	2.89	0.007
		Bachelor’s degree and above		1.24 (0.62, 1, 94)	0.32	4.55	< 0.001
**Panel B**							
Tobacco and alcohol use	Sex	Female	Male	0.33 (0.13, 0.87)	0.27	3.17	0.001
	Education	High school	Junior secondary school and below	1.53 (0.59, 2.57)	0.49	3.31	< 0.001
		Bachelor’s degree and above		1.91 (2.12, 3.25)	0.62	3.26	< 0.001
Physical activity	Age	80 and above	60–80	-1.32 (-1.94, -0.67)	0.33	-3.91	< 0.001
	Marriage	Single/divorced/widowed	Married	-1.13 (-1.67, -0.35)	0.38	-2.96	0.002
Stress management	Age	80 and above	60–80	-0.53 (-0.86, -0.17)	0.21	-2.88	0.007
	Income	Middle	Lower-middle	1.03 (0.44, 1.68)	0.32	3.75	< 0.001
		Upper-middle		0.86 (0.30, 1.72)	0.37	3.49	< 0.001
Health responsibility	Income	Middle	Lower-middle	1.31 (0.20, 2.36)	0.52	2.46	0.018
		Upper-middle		1.87 (0.74, 3.11)	0.71	2.94	0.003
	Education	High school	Junior secondary school and below	1.65 (0.67, 2.54)	0.48	3.41	< 0.001
		Bachelor’s degree and above		1.43 (0.51, 2.39)	0.46	3.34	< 0.001
**Panel C**							
Spiritual growth	Income	Middle	Lower-middle	1.23 (0.26, 2.12)	0.50	2.32	0.024
		Upper-middle		1.75 (0.57, 3.07)	0.67	3.28	< 0.001
	Age	80 and above	60–80	-0.59 (-1.01, -0.07)	0.21	-2.56	0.013
	Marriage	Single/divorced/widowed	Married	-1.05 (-1.65, -0.23)	0.33	-2.73	0.009
	Education	High school	Junior secondary school and below	0.97 (0.38, 1.61)	0.30	3.18	0.001
		Bachelor’s degree and above		1.29 (0.87, 2.36)	0.34	3.98	< 0.001

Only statistically significant (*P* < 0.05) independent variables are listed in Table 5

## Conclusion & discussion

### Overall respondents exhibited good health behaviour

Overall, elderly patients with NCDs in Jiangsu Province behaved in a moderately healthy manner. Specifically, the average standardised total health-behaviour score was 76.73, which indicates that the respondents exhibited “good” health behaviour. There are three explanations for this optimistic result. First, public healthcare services for elderly citizens are relatively well-developed in Jiangsu Province. For example, in the annual rankings released by the National Health Committee in 2019, Jiangsu Province ranked 3/31 in terms of basic public healthcare services. Moreover, one-third of the nursing homes in the country are located in Jiangsu Province. Second, health education for the general public has been promoted in recent years. For example, as stated by the National Health Committee in 2019, 25.33% of residents in Jiangsu Province qualified for health literacy (including basic health knowledge, healthy lifestyle, and basic skills); this proportion was higher than that of the average proportion of the whole country (19.17%). Additionally, the sanitary behaviours and health education promoted during the COVID-19 pandemic have raised public awareness of healthy behaviours. Third, due to their NCDs, patients tend to frequently visit doctors (for physical examinations or medical treatment) and therefore are more likely to receive health advice from professionals and to improve their behaviour accordingly.

### Differences across the dimensions of health behaviour

Upon deeper examination of health behaviours, we noticed differences in scores across the dimensions. Specifically, respondents scored highest on the dimension of “nutrition” (82.63 ± 8.92, standardised), followed by “tobacco and alcohol use”, “health responsibility”, “spiritual growth”, “stress management”, and “physical activity” (70.17 ± 10.25, standardised), in line with Huy, Schneider, and Thiel [14]. The high score of respondents on “nutrition” was interpreted as follows. First, nutrition generally improves with economic growth and nutrition education for the public. Second, as mentioned above, patients with NCDs are more likely to receive advice concerning nutrition from medical professionals during their regular treatment or physical examinations. Third, nutrition (e.g., increased intake of protein) and sanitary food practices (e.g., avoiding uncooked food) were emphasised in health education during the COVID-19 pandemic.

Additionally, respondents’ low score on “physical activity” is reasonable. First, respondents in this survey generally suffered from impaired physical performance because of age and disease. Second, since the majority of respondents lived in urban areas where stairs are impossible to avoid, some of them avoided going outside due to fear of a fall. Third, the survey was conducted during the COVID-19 pandemic; thus, elderly residents were encouraged to avoid crowds.

Respondents scored second and third highest on “tobacco and alcohol use” (77.13 ± 12.63, standardised) and “health responsibility” (76.15 ± 12.85, standardised), respectively, in line with Holford et al. [13] and Kerr, Greenfield, Bond, Ye, and Rehm [18]. In other words, elderly patients with NCDs exhibited moderately healthy behaviours on items that concerned self-discipline, possibly due to the abovementioned professional health advice they received. The comparatively lower scores of participants on “spiritual growth” (75.25 ± 13.25, standardised) and “stress management” (75.20 ± 10.15, standardised), in line with Cornwell [6] and Jackson, Knight, and Rafferty [15], indicate a lower goals and desires in this ageing population and decreased happiness because of age and disease.

### Demographics as determinants of health behaviour

Typically, married respondents less than 80 years old with higher education and upper-middle income scored higher on health behaviour. First, we found that age affects health behaviour, possibly due to limited physical abilities. However, age does not affect all six dimensions of health behaviour equally (e.g., the impact of age on tobacco and alcohol use is nonsignificant).

Second, we showed that education has a positive effect on health behaviour, in line with Conti, Heckman, and Urzua [5]. Specifically, better-educated respondents are more likely to score higher in terms of “nutrition”, “tobacco and alcohol use”, “health responsibility”, and “spiritual growth”. Generally, better-educated individuals are aware of their physical issues and behave in a manner in accordance with scientific guidelines. For example, better-educated respondents are more aware of their NCDs and follow their doctors’ advice as to diet and abstaining from tobacco and alcohol use.

Third, we found that income has a positive effect on health behaviour, in line with Deaton [8] and Stronks, Van De Mheen, Van den Bos, and Mackenbach [30]. Specifically, respondents with upper-middle income levels are more likely to score higher on “stress management”, “health responsibility”, and “spiritual growth”. According to the theory of self-efficacy [1], income affects health behaviour through its indirect influence on emotions and through the residential environment. Respondents with higher income levels are more likely to live in neighbourhoods with better healthcare services and physical infrastructures (e.g., gyms). In addition, individuals with higher income levels generally enjoy more leisure time, which enables them to participate in social activities and maintain healthy relationships.

Fourth, our results indicated that marriage has a positive effect on health behaviour, in line with Burgard, Lin, Segal, Elliott, and Seelye [3]. Specifically, we found that married respondents were more likely to score higher on “physical activity” and “spiritual growth”. According to the theories of social support [35] and family systems [11], married individuals are more likely to improve their health behaviour through communicating with their spouse. In contrast, single/divorced/widowed individuals are more likely to be lonely and pessimistic, which might undermine their health behaviour.

Therefore, the results imply that single/divorced/widowed elderly patients more than 80 years old with less education and lower income levels are the group typically in need of health interventions. Furthermore, we found that the dimensions of “stress management” and “physical activity” should be the focus of the health interventions. Specifically, we recommend that healthcare infrastructure be designed to target the abovementioned group for health intervention. Additionally, while physical activity is critical in preventing disease in elderly people [31], we found that physical performance declines with age. In addition, the abuse of substances (e.g., smoking and excessive alcohol consumption) has a lifetime cumulative effect because of addiction [12][17] and increases mortality in old age [19][32]. Therefore, health education is recommended as a preventive intervention for residents starting at a young age. In a broader sense, our data provide further global implications. The results above show that income improves health behaviour, and substantial evidence indicates that health behaviour is crucial for treating NCD [2]. Thus, income levels may be responsible for the fact that adults in low- (21%) and lower-middle-income (23%) economies suffer twice as much risk of NCD as high-income economies [37]. This implies that economic growth and income allocation are dominant health behaviours at the national level that can even influence mortality in the long term.

### Limitations & future directions

A major limitation of this research is its biased sampling methods. First, the bias towards individuals with middle- and upper-middle income levels is obvious due to the relatively small proportion of low-income individuals in the sample, although this proportion is consistent with the income of the entire population of the province. The disposable per capita income of Jiangsu ranks fifth among the 32 province-level areas in the country. In addition, most surveys were conducted in parks where elderly residents work out and socialise. Generally, residents with leisure time for exercise and entertainment are better off. Second, neither sex or NCD category significantly impacted health behaviour, which might indicate sampling bias. Thus, in future studies, the sampling method needs to be optimised to capture broader strata. Third, our sample excluded people without NCDs and those suffering from diseases that prevent communication. As mentioned above, patients with NCDs are characterised by regular doctor visits and opportunities to receive medical advice. In future research, people without NCD could be included as a control group to identify the effect of medical advice on health behaviour. Moreover, in-depth interviews with mute patients, patients with dementia or Parkinson’s disease or their long-term caregivers could be carried out with the aid of technology to explore the health behaviour of elderly residents with NCD and more complicated health scenarios.

Another major limitation of this research is that the survey focused on “downstream” (i.e., individual) determinants of health behaviour but neglected “upstream” (i.e., social) determinants [25]. Therefore, in future studies we plan to include external determinants (e.g., health insurance, physical infrastructure, and online health education).

## Data Availability

The data that support the findings of this study are available from the corresponding author, Y.G., upon reasonable request.

## Footnotes

1 In mainland China, one prefecture includes several districts and counties. Compared with that of the districts, the local government of the counties enjoy greater autonomy and the proportion of rural residents are usually higher.

2 Investigators spoke to the respondents in person or on phone. A caregiver was required to be present for assistance. Excluded samples were those participants whose caregiver refused the survey on their behalf and stated that they suffered from muteness, dementia or Parkinson’s disease. In this case, such a sample would be replaced by another randomly selected one.

3 If the respondent was not a chronic disease patient, the “chronic disease category” in his or her questionnaire was left blank. Thus, a total of 132 respondents that reported they were free of chronic disease were excluded.
